# Fabp4-CreER lineage tracing revealstwo distinctive coronary vascular populations

**DOI:** 10.1111/jcmm.12415

**Published:** 2014-09-30

**Authors:** Lingjuan He, Xueying Tian, Hui Zhang, Joshua D Wythe, Bin Zhou

**Affiliations:** a Key Laboratory of Nutrition and Metabolism, Institute for Nutritional Sciences, Shanghai Institutes for Biological Sciences, Graduate School of the Chinese Academy of Sciences, Chinese Academy of SciencesShanghai, China; b Cardiovascular Research Institute, Department of Molecular Physiology and Biophysics, Baylor College of MedicineHouston, TX, USA

**Keywords:** coronary vessel, angiogenesis, coronary artery diseases, heart regeneration

## Abstract

Over the last two decades, genetic lineage tracing has allowed for the elucidation of the cellular origins and fates during both embryogenesis and in pathological settings in adults. Recent lineage tracing studies using Apln-CreER tool indicated that a large number of post-natal coronary vessels do not form from pre-existing vessels. Instead, they form *de novo* after birth, which represents a coronary vascular population (CVP) distinct from the pre-existing one. Herein, we present new coronary vasculature lineage tracing results using a novel tool, Fabp4-CreER. Our results confirm the distinct existence of two unique CVPs. The 1^st^ CVP, which is labelled by Fabp4-CreER, arises through angiogenic sprouting of pre-existing vessels established during early embryogenesis. The 2^nd^ CVP is not labelled by Fabp4, suggesting that these vessels form *de novo*, rather than through expansion of the 1^st^ CVP. These results support the *de novo* formation of vessels in the post-natal heart, which has implications for studies in cardiovascular disease and heart regeneration.

## Introduction

Coronary artery disease is the leading cause of mortality and morbidity in the western world 1–3. Atherosclerotic blockage or injury of the coronary arteries impairs the delivery of oxygen and vital nutrients to the myocardium, potentially inducing myocardial infarction and cardiomyocytes death. There exists an urgent, unmet need for strategies that stimulate the formation of new vessels within the injured heart muscle. At the most basic level, knowledge of how the mammalian heart coronary vascular tree is established during embryonic development is an essential prerequisite for understanding this disease, and it may also suggest new therapeutic strategies to combat the main cause of death worldwide 4.

Advances in genetic lineage tracing based on Cre–loxP recombination in mice have led to a recent reassessment of not only the origin of the coronary arteries but also the developmental process of coronary vascular tree formation 5–7. The cellular origin(s) of the coronary arteries remains highly controversial, with recent studies identifying at least three major distinct sources: the (pro)epicardium, the endocardium of the sinus venosus, and the ventricular endocardium 8–10. Recent lineage tracing studies using an Apln-CreER allele revealed that early embryonic vessels do not contribute to the majority of new vessels in the post-natal heart. In fact, post-natal new vessels appear to form *de novo* through lineage conversion from non-vascular cells 11, thus modifying the previous angiogenic paradigm explaining post-natal coronary vessel addition.

## Material and Methods

This study was carried out in strict accordance with the recommendations in the Guide for the Care and Use of Laboratory Animals of the Chinese Academy of Sciences. The protocol was approved by the Institutional Animal Care and Use Committee of the Institute for Nutritional Sciences, Shanghai Institutes for Biological Sciences. Tie2-Cre, Fabp4-Cre, Fabp4-CreER, Rosa26^mTmG/+^ and Rosa26^RFP/+^ mice were described previously 12–16. Female mice were mated with adult males overnight and then examined the female mice in following morning for the presence of a vaginal plug as embryonic day 0.5 (E0.5). Immunostaining was performed according to protocols described previously 11.

## Results and discussion

**Figure 1:**
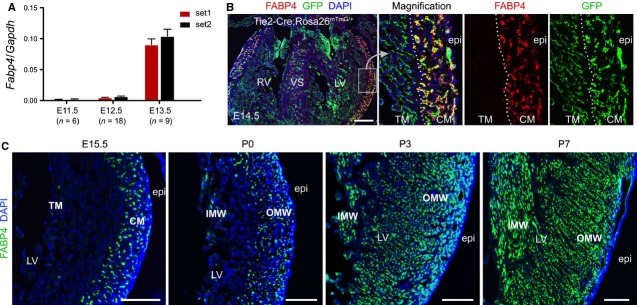
FABP4 expression in embryonic and neonatal heart. (**A**) Relative Fabp4 mRNA expression in developing hearts shows its expression increases at E13.5. Two different sets of primers were used in qRT-PCR analysis. (**B**) Immunostaining of FABP4, GFP and DAPI on E14.5 Tie2-Cre; Rosa26^mTmG/+^ heart sections. White dotted line delineates the border between trabecular myocardium (TM) and compaction myocardium (CM). While GFP labels both endocardial cells in TM and coronary vascular endothelial cells (VECs) in CM, FABP4 specifically marks VECs in CM, but not endocardial cells in TM. RV, right ventricle; VS, ventricular septum; LV, left ventricle; epi, epicardium. (**C**) Immunostaining of FABP4 and DAPI on E15.5, P0, P3 and P7 heart sections. Images are representative of four individual samples for each stages; white bar = 200 μm. OMW, outer myocardial wall; IMW, inner myocardial wall.

To verify and extend this critical finding, we focused our attention on a second gene, fatty acid binding protein 4 (Fabp4) 12. Interestingly, Fabp4 is detected specifically in coronary vascular endothelial cells (VECs) in the adult heart 17. As an initial first step, we confirmed this restricted pattern of FABP4 expression. Quantitative RT-PCR of embryonic hearts showed that *Fapb4* expression was significantly increased at E13.5, a stage when intramyocardial coronary vessels form and expand (Fig. 1A). While a pan-endothelial Cre line (Tie2-Cre) labelled both coronary VECs and endocardial cells in E14.5 hearts, FABP4 was detected only in coronary VECs in the compact myocardium, and notably absent in endocardial cells of the trabecular myocardium (TM; Fig. 1B). There are few FABP4^+^ coronary vessels in the TM or inner myocardial wall (IMW) at E15.5 and P0. However, at P3 and P7, a significant amount of FABP4^+^ vessels arise in IMW (Fig. 1C), indicating the new addition of coronary vessels in the neonatal heart.

**Figure 2:**
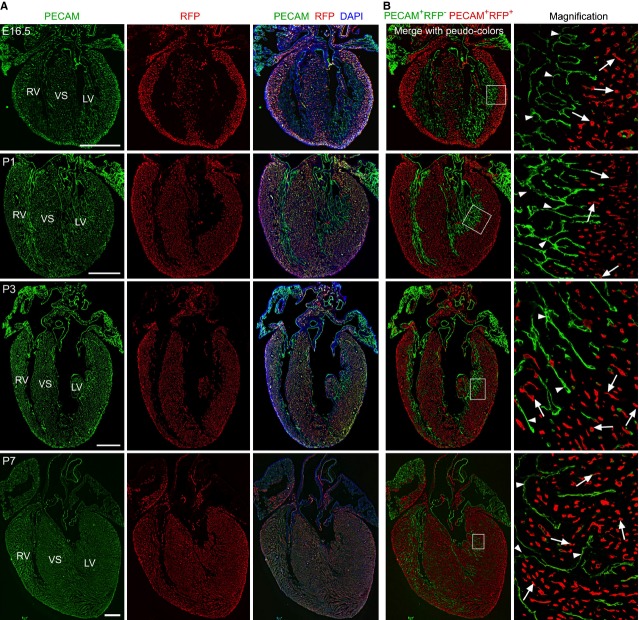
Lineage tracing of coronary endothelial cells by Fabp4-Cre; Rosa26^RFP^^/+^ line. (**A**) Immunostaining of PECAM, RFP and DAPI on E16.5, P1, P3, P7 Fabp4-Cre; Rosa26^RFP^^/+^ line. (**B**) For contrast, pictures in A were pseudo-coloured by green fluorescence for PECAM+RFP cells (arrowheads in magnified pictures) and red fluorescence for PECAM^+^RFP^+^ cells (white arrows in magnified pictures); white bar = 500 μm. LV, left ventricle; RV, right ventricle; VS, ventricular septum. Images are representative of four individual samples for each stages.

We next leveraged this unique FABP4 expression pattern and performed lineage tracing in Fabp4-Cre; Rosa26^RFP/+^ mice 13,14. Cre–loxP-mediated genetic lineage tracing is both heritable and irreversible, permanently identifying the descendants by their expression of the genetic reporter (*e.g*. RFP). By collecting hearts at embryonic day 16.5 (E16.5), post-natal day 1 (P1), P3 and P7, we found that Fabp4-Cre specifically labelled coronary VECs, but not endocardial cells at each of these stages (Fig. 2), reinforcing the unique and tissue-specific pattern of FABP4 expression in the coronary VECs.

**Figure 3:**
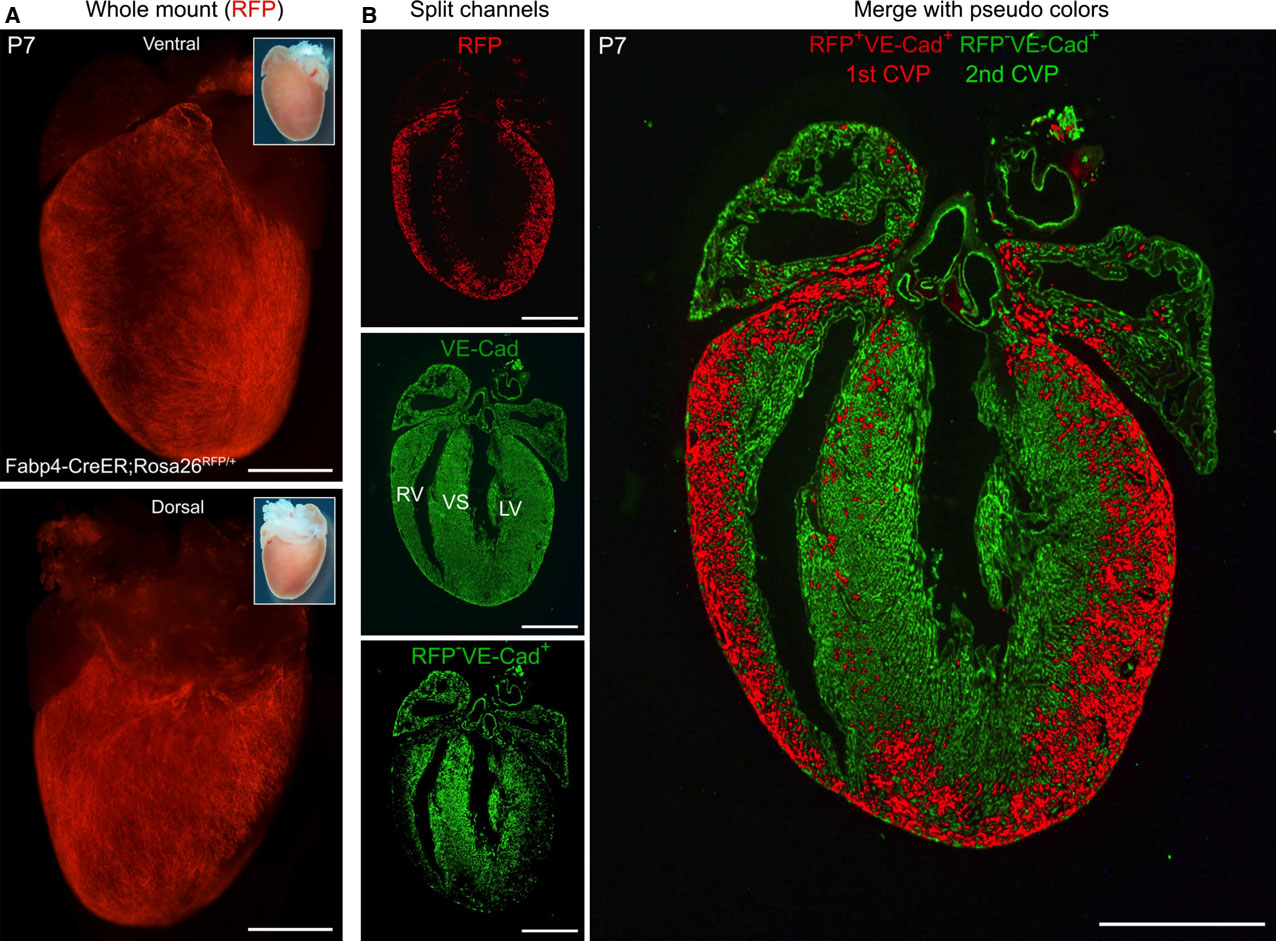
Images of two coronary vascular populations distinguished by Fabp4-CreER lineage tracing. (**A**) Whole mount phase contrast and fluorescence view of P7 Fabp4-CreER; Rosa26^RFP^^/+^ heart. Tamoxifen was induced at E13.5. (**B**) Immunostaining of lineage tracing marker RFP, endothelial cell marker VE-Cadherin (VE-Cad) on sections of P7 Fabp4-CreER; Rosa26^RFP^^/+^ heart. On the right panel, RFP+VE-Cad+ cells are termed 1^st^ coronary vascular population (1^st^ CVP) and shown by pseudo-colour red, while RFP-VE-Cad+ cells are termed 2^nd^ CVP and shown by pseudo-colour green; white bar = 1 mm. Representative images of three individual samples. LV, left ventricle; RV, right ventricle; VS, ventricular septum.

While informative for some studies, the constitutive nature of the Fabp4-Cre alleles does not provide critical information about whether these new vessels arise from pre-existing vessels or form *de novo* from non-vascular sources. We therefore employed an inducible Cre allele for ‘pulse-chase’ experiments to determine the contribution of embryonic vessels to the post-natal coronary vasculature. Translocation of Cre recombinase fused to a mutant oestrogen receptor (CreER) into nucleus is a prerequisite for combination of loxP sites. This translocation of CreER depends on the exogenous addition of the synthetic oestrogen receptor ligand tamoxifen at a desired time-point (‘pulse’), which allows for the indelible labelling and tracing of cellular fates and contributions during development (‘chase’). Based on the unique and specific expression of FABP4 (Fig. 1), we performed inducible genetic lineage tracing in Fabp4-CreER; Rosa26^RFP/+^ mice 12,14. Pregnant dams were pulsed with tamoxifen during embryogenesis (E13.5-14.5) to label the early coronary vessels, and we then examined the contribution of these early-labelled vessels and their derivatives (RFP^+^, tracer) to the P7 neonatal hearts. Whole mount view of P7 Fabp4-CreER; Rosa26^RFP/+^ hearts revealed that RFP^+^ cells covered the surface of the heart (Fig. 3A). However, immunostained sections of the same hearts showed that a large portion of coronary VECs within the myocardium were actually not labelled (*i.e*. they were RFP^−^). As Cre–loxP-mediated genetic lineage tracing is irreversible and heritable, the genetic labelling (RFP) would inevitably label all descendants of these early FABP4^+^ embryonic VECs, regardless of whether they express CreER at P7 or not. Therefore, these RFP^−^ vessels in the inner layer of myocardium could not be derivatives of the FABP^+^ embryonic VECs, suggesting *de novo* formation of coronary VECs in the post-natal heart. Endocardial cells, which form the innermost layer of heart, and are adjacent to the myocardium, could be a potential progenitor pool for coronary vascular cells 11, as well as other cell types such as mesenchymal valve cells, intramyocardial fibroblasts and the recently discovered telocytes 18–23.

Significantly, combined immunostaining of the Fabp4 lineage (RFP^+^) and endothelial cells (Ve-cadherin, VE-Cad) in post-natal Fabp4-CreER; Rosa^RFP/+^ hearts (Fig. 3B) demonstrated that two unique, non-overlapping populations of cells contribute to the coronary vasculature. The first population of cells, or 1^st^ CVP, are derived from the Fabp4-CreER embryonic lineage and are RFP^+^; Ve-cadherin^+^, while those of the 2^nd^ CVP are not marked by Fabp-CreER (RFP^−^; Ve-cadherin^+^) and are derived *de novo*. Understanding how this 2^nd^ CVP forms would greatly aid studies of cardiovascular diseases, such as myocardial non-compaction and myocardial regenerative medicine.
